# Restoring Microbial Signaling: A Metabolite–Immune–Redox Framework for Postbiotic Host-Directed Interventions

**DOI:** 10.3390/medsci14040438

**Published:** 2026-07-26

**Authors:** Dejana Bajić, Nemanja Todorović, Mladena Lalić Popović, Jelena Vučković, Andrea Mihajlović, Danijel Slavić, Borislav Tapavički, Mirjana Stojšić, Nataša Milošević

**Affiliations:** 1 Department of Biochemistry, Faculty of Medicine, University of Novi Sad, 21000 Novi Sad, Serbia; 2 Department of Pharmacy, Faculty of Medicine, University of Novi Sad, 21000 Novi Sad, Serbia; nemanja.todorovic@mf.uns.ac.rs (N.T.); mladena.lalic-popovic@mf.uns.ac.rs (M.L.P.); natasa.milosevic@mf.uns.ac.rs (N.M.); 3 Centre for Medical and Pharmaceutical Investigations and Quality Control (CEMPhIC), Faculty of Medicine, University of Novi Sad, 21000 Novi Sad, Serbia; 4 Department of Anesthesiology and Perioperative Medicine, Faculty of Medicine, University of Novi Sad, 21000 Novi Sad, Serbia; jelena.vuckovic@mf.uns.ac.rs; 5 Institute for Cardiovascular Diseases of Vojvodina, Put Doktora Goldmana 4, 21204 Sremska Kamenica, Serbia; 6 Department of Physiology, Faculty of Medicine, University of Novi Sad, 21000 Novi Sad, Serbia; andrea.zubnar@mf.uns.ac.rs (A.M.); danijel.slavic@mf.uns.ac.rs (D.S.); borislav.tapavicki@mf.uns.ac.rs (B.T.); 7 Department of Pediatrics, Faculty of Medicine, University of Novi Sad, 21000 Novi Sad, Serbia; mirjana.stojsic@mf.uns.ac.rs; 8 Department of Gastroenterology, Hepatology and Nutrition, Institute for Child and Youth Health Care of Vojvodina, 21000 Novi Sad, Serbia

**Keywords:** postbiotics, gastrointestinal microbiome, microbial metabolites, extracellular vesicles, oxidative stress, precision medicine, host-directed therapy

## Abstract

**Background/Objectives:** Postbiotics are increasingly recognized as biologically active products of microorganisms with emerging potential as microbiome-inspired therapeutic interventions. While most microbiome-based strategies focus on modifying microbial composition, restoration of microbial signaling has received comparatively less attention. This review examines postbiotics through the lens of microbial signaling restoration and proposes a unified Metabolite–Immune–Redox (MIR) axis linking microbial-derived signals with immune regulation, redox homeostasis, endothelial integrity, and host resilience. **Methods:** This narrative review synthesizes current evidence on postbiotics, microbial metabolites, structural microbial components, and extracellular vesicles, with emphasis on their roles in immunometabolic regulation, redox biology, endothelial function, and host-directed interventions. **Results:** Current evidence suggests that short-chain fatty acids, indole derivatives, bile acid metabolites, and microbial extracellular vesicles are important mediators of host–microbe communication. These signals influence interconnected pathways involving mitochondrial function, inflammasome activity, immune calibration, endothelial and glycocalyx homeostasis, and disease tolerance. The review highlights the endothelium as an underrecognized therapeutic target and discusses biomarkers, including soluble thrombomodulin, von Willebrand factor, and D-dimer, as potential tools for identifying patients most likely to benefit from host-directed interventions. Major translational challenges include product heterogeneity, incomplete mechanistic characterization, uncertain exposure–response relationships, and unresolved regulatory considerations. **Conclusions:** The proposed MIR axis provides a hypothesis-generating framework for understanding how restoration of microbial signaling may contribute to precision host-directed therapeutic strategies. Further mechanistic and clinical studies are needed to validate this concept and define its translational potential in inflammatory, infectious, and critical illness settings.

## 1. Introduction

Despite remarkable progress in microbiome research, translating these discoveries into mechanism-driven therapeutics remains challenging [1,2]. While many microbiome-based strategies have traditionally focused on modifying microbial composition, increasing attention is now being directed toward understanding microbial functional activity and its contribution to host physiology, recognizing that microbial composition and function are fundamentally interconnected [1,2,3,4,5]. Indeed, alterations in microbial community structure do not necessarily translate into restoration of the biochemical signaling networks through which the microbiome regulates host physiology [5,6,7]. Consequently, growing emphasis has been placed not only on correcting dysbiosis, but also on restoring microbial-derived signaling pathways that contribute to immune, metabolic, and redox homeostasis [4,5,6,7].

This functional view carries important therapeutic implications. The depletion or distortion of bioactive microbial signals—including short-chain fatty acids, indole derivatives, bile acid metabolites, and microbial extracellular vesicle cargo—may contribute to disease not merely through altered microbial ecology, but through loss of protective pathways that ordinarily buffer inflammation, sustain endothelial integrity, and preserve adaptive responses under stress [4,5,6,7]. Emerging evidence further suggests that certain bacteriocins may also interact directly with host cells through immunomodulatory and signaling mechanisms, extending their biological role beyond antimicrobial activity [8,9]. In this sense, pathology may reflect a deficit of host-protective signaling as much as a burden of harmful stimuli [1,4,5]. Such a reframing complements ecological perspectives on microbiota composition by emphasizing microbial-derived signaling as an additional therapeutic dimension [1,3,5] and raises a more pharmacologically relevant question: can the restoration of microbial-derived signaling itself become a therapeutic strategy? In this context, the restoration of microbial signaling may represent a therapeutically actionable concept capable of bridging microbiome science, redox pharmacology, endothelial biology, and precision medicine [4,5,6,7,10,11].

Importantly, restoration of microbial-derived signaling should not be interpreted as a substitute for restoration of a healthy and functionally resilient microbiota [1,3,5]. Rather, microbial signaling may represent one mechanistic component of a broader recovery process, in which sustained therapeutic benefit ultimately depends on the re-establishment of microbial ecological stability and functional host–microbiota interactions [3,5]. In this context, postbiotics may serve as complementary host-directed interventions that reinforce protective signaling pathways while facilitating the gradual restoration of endogenous microbial functional capacity and overall microbiota resilience [2,3,8,9].

Throughout this review, the term “microbiome-derived” is used to describe microbial signaling processes and functional products involved in host–microbe communication within the proposed MIR framework, rather than to imply that all postbiotic preparations necessarily originate from microorganisms belonging to the human microbiome [10].

Within this emerging paradigm, postbiotics have attracted growing interest because they may provide a route from microbiome modulation toward defined functional intervention. Unlike strategies dependent on live microbial engraftment, postbiotics offer the conceptual advantage of delivering microbial bioactivity in a form potentially more compatible with standardization, dose control, and translational development [2,10,11]. This has begun to position them less as extensions of probiotic biology and more as candidate postbiotic therapeutic interventions with drug-like properties. Viewed through this lens, postbiotics may be interpreted as next-generation pharmabiotics whose significance lies not simply in their microbial origin, but in their capacity to engage identifiable host pathways relevant to disease modification [12,13]. However, whether postbiotics exert direct disease-modifying effects or primarily serve as markers of broader microbiome-associated health remains an area of ongoing debate. This distinction is particularly important when considering their potential development as mechanistically defined therapeutic agents.

**Figure 1:**
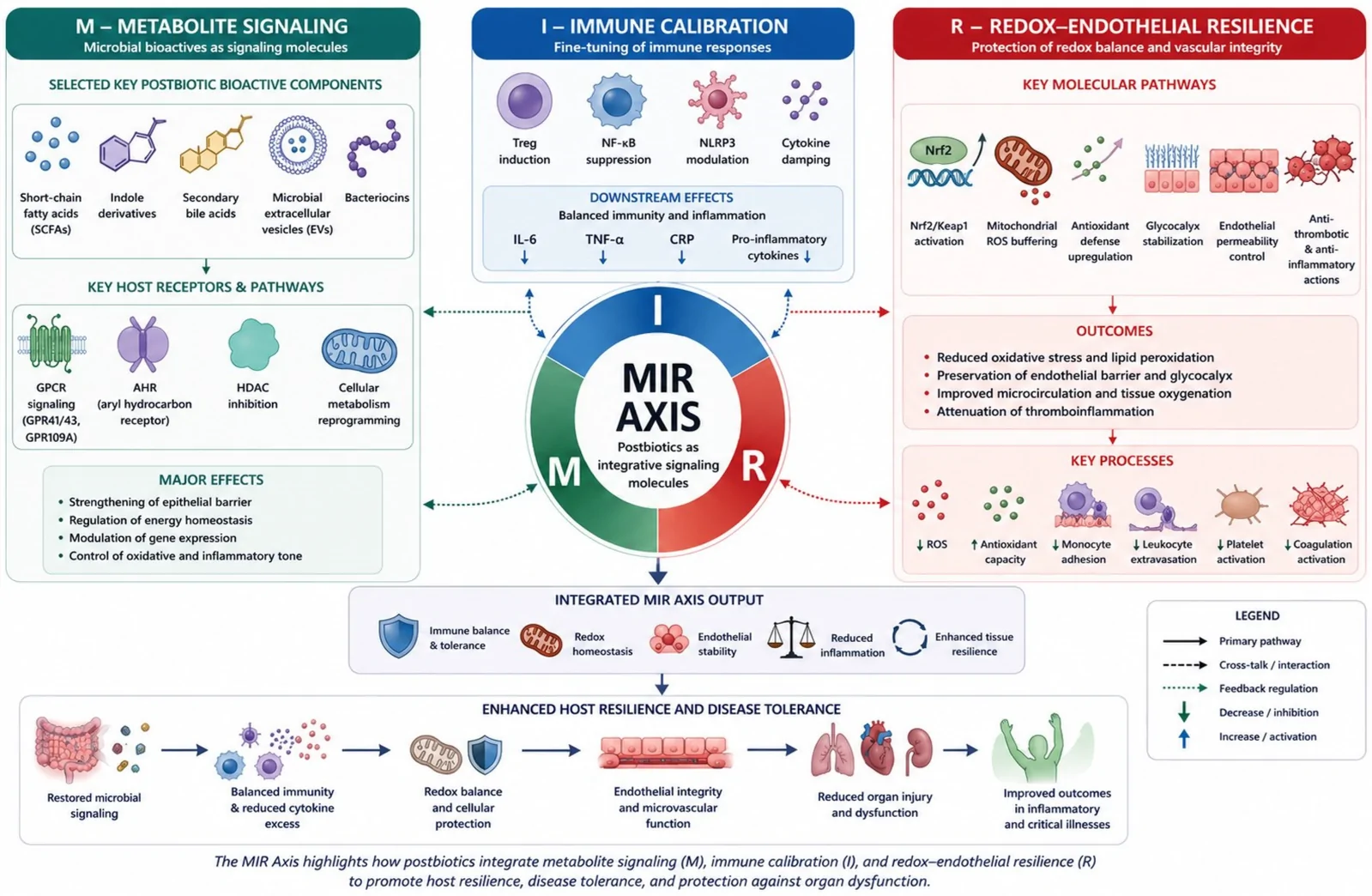
The MIR Axis: a conceptual framework integrating postbiotic bioactive components with metabolite signaling, immune calibration, and redox–endothelial resilience. The MIR Axis illustrates a hypothesis-generating conceptual framework in which selected bioactive components of postbiotics—including microbial metabolites, extracellular vesicles, and bacteriocins—contribute to metabolite signaling (M), immune calibration (I), and redox–endothelial resilience (R), thereby supporting host resilience, disease tolerance, and protection against organ dysfunction. Colored dashed arrows illustrate the conceptual cross-talk between the metabolite signaling (M), immune calibration (I), and redox–endothelial resilience (R) modules, whereas solid arrows represent the principal biological pathways within each module.

Here we propose that this therapeutic potential may be understood through a unified Metabolite–Immune–Redox (MIR) axis, in which microbial-derived signals converge on three interconnected domains: metabolic signaling, immune calibration, and redox–endothelial resilience (Figure 1) [4,5]. The MIR framework should be viewed as a hypothesis-generating conceptual model that integrates emerging evidence across multiple biological systems and is intended to guide future mechanistic and clinical investigation. Importantly, the MIR axis is not intended to replace existing frameworks such as the Gut–Brain Axis, immunometabolism, or host-directed therapy models, but rather to integrate these perspectives into a unified and testable conceptual framework. This concept extends beyond classical immunomodulation by suggesting that postbiotics may represent candidates for precision redox-oriented therapeutics, with the potential to reinforce endogenous pathways involved in oxidative stress adaptation, inflammatory set-point control, and vascular homeostasis [14,15] (Table 1). Such a view is particularly relevant in inflammatory and critical illness settings, where failure of resilience rather than pathogen burden alone often drives progression toward organ dysfunction [16].

This perspective also aligns with evolving concepts in host-directed therapy, where therapeutic value is increasingly linked not solely to suppressing pathogenic processes, but to strengthening host capacity to tolerate injury [17]. In that context, host resilience can be understood as the integrated ability to preserve function through maintenance of redox balance, immune calibration, and endothelial stability under stress, while disease tolerance reflects the capacity to limit tissue injury without necessarily altering pathogen load [18]. Interpreted this way, postbiotics may represent candidate enhancers of both resilience and disease tolerance, acting through restoration of protective signaling rather than direct antimicrobial activity [5,14,16].

An additional conceptual dimension emerges from hormesis, whereby low-intensity biological signals activate adaptive stress responses that enhance system-level robustness [19,20]. Some postbiotic effects may plausibly operate through such mechanisms, functioning not merely as passive metabolites but as signaling cues capable of triggering cytoprotective redox programs, mitochondrial adaptation, and restraint of self-amplifying inflammatory injury [20,21,22]. This possibility is especially compelling when considered alongside emerging evidence implicating oxidative stress, endothelial dysfunction, and thromboinflammation as shared drivers across sepsis, viral critical illness, and chronic inflammatory disorders [4,14,22].

Taken together, these considerations support a broader pharmacological hypothesis: postbiotics should not be viewed solely as successors to probiotics or synbiotics, but rather as a distinct class of postbiotic host-directed interventions based on defined microbial bioactive components that engage conserved host signaling pathways and may have applications across multiple biomedical and clinical contexts (Table 2) [23,24,25,26,27]. On this basis, this review examines the proposition that postbiotics may represent an emerging platform for pharmacologic interventions based on microbial bioactive components. Throughout this review, the term “microbiome-derived” is used to refer to microbial signaling molecules and functional products that mediate host–microbe communication, rather than to imply that all postbiotic preparations necessarily originate from the human microbiome [13,23,24,25,26,27].

The aim of this review is to synthesize current evidence on postbiotics within a unified Metabolite–Immune–Redox (MIR) framework and to evaluate the potential of postbiotic host-directed interventions within the proposed MIR framework. By integrating emerging findings from microbial metabolite biology, extracellular vesicle research, redox regulation, endothelial resilience, and precision medicine, this review seeks to provide a translational perspective on microbial signaling restoration.

Collectively, the available evidence supports the view that postbiotics may provide a rational platform for future biomarker-guided therapeutic strategies. Unlike many probiotic interventions, whose clinical translation is often limited by the inability to directly quantify or standardize their functional bioactive signals in vivo, postbiotic preparations contain defined microbial bioactive components that can be chemically characterized, quantified, and linked to measurable host pharmacodynamic biomarkers. Establishing this functional relationship between postbiotic composition and host biomarker responses may represent one of the key prerequisites for biomarker-guided microbiome-based therapeutic strategies. Nevertheless, substantial mechanistic and clinical validation remains necessary before such approaches can be implemented in routine clinical practice [2,10,11,14,15].

In particular, future clinical studies should evaluate not only clinical outcomes, but also the quantitative relationship between defined postbiotic bioactive components and host pharmacodynamic biomarker responses, thereby establishing quantitative exposure–biomarker–response relationships analogous to those routinely employed in translational pharmacology for pharmacodynamic characterization and biomarker-guided therapeutic development. Such an approach may facilitate biomarker-guided patient stratification, improve mechanistic understanding of treatment response, and support the rational development of standardized postbiotic interventions [10,11,14,15].

### Literature Search and Scope of the Review

This review was designed as a structured narrative review to summarize current evidence on postbiotics and microbial signaling and to integrate mechanistic, translational, and clinical perspectives relevant to the proposed MIR framework. Because the objective was to synthesize multiple interconnected biological concepts rather than address a single focused clinical question, a narrative approach was considered the most appropriate.

The literature search was performed using PubMed and Web of Science Core Collection from database inception through June 2026. Search terms included combinations of postbiotics, microbial metabolites, extracellular vesicles, microbial signaling, short-chain fatty acids, indole derivatives, redox biology, immune regulation, endothelial dysfunction, host-directed therapy, critical illness, and precision medicine. Additional relevant publications were identified through manual screening of reference lists from key articles and recent reviews.

Priority was given to consensus statements, mechanistic studies, translational research, and clinically relevant human studies. Historical publications were included selectively when necessary to provide scientific context or illustrate the evolution of concepts discussed in this review.

## 2. Postbiotics as Redox Therapeutics

A particularly important conceptual shift in the field has been the recognition that some postbiotic effects may be interpreted not only through immunomodulation, but through redox biology. This distinction matters because oxidative stress in inflammatory disease is increasingly viewed less as a simple excess of reactive oxygen species and more as a failure of adaptive redox regulation [4,30]. Within this framework, microbial metabolites are relevant not merely because they dampen inflammatory signaling, but because they may reinforce endogenous pathways that preserve oxidative homeostasis. This perspective has drawn particular attention to short-chain fatty acids, especially butyrate, whose biological relevance appears to extend well beyond gut barrier maintenance. Emerging evidence suggests that butyrate may influence Nrf2-linked cytoprotective responses, support mitochondrial efficiency, and redirect inflammatory metabolism away from oxidative injury toward adaptive resilience [6,20,31]. The implication is significant: if some postbiotics reinforce host antioxidant programs rather than merely counter oxidative stress secondarily, they begin to resemble redox-active signaling therapeutics rather than conventional nutritional adjuncts [21,31,32].

A parallel conceptual expansion is emerging around indole derivatives and the aryl hydrocarbon receptor (AhR), where interest has shifted from mucosal tolerance alone toward the intersection of barrier biology, immune calibration, and redox control [6,33]. In this context, immune calibration denotes something more precise than generalized immunomodulation; it refers to fine-tuning inflammatory and regulatory pathways toward controlled host defense with limited collateral tissue injury [4,14,34]. That distinction is important because the biological goal is not indiscriminate suppression of inflammation, but optimization of inflammatory set-points compatible with tissue protection and disease tolerance [18,35]. Indole signaling is increasingly relevant in this regard because epithelial integrity and oxidative homeostasis appear mechanistically intertwined: barrier disruption can amplify inflammatory ROS signaling, while impaired redox control further destabilizes barrier function. Interpreted through this feedback logic, AhR-active microbial metabolites may help stabilize both systems simultaneously [36,37]. This is also one reason NLRP3 attenuation has emerged as a mechanistically important theme, since inflammasome activation occupies a convergence point between mitochondrial stress, oxidative injury, and inflammatory amplification [4,6,33]. The broader interpretation is that some postbiotics may interrupt self-reinforcing redox–inflammasome loops that propagate tissue damage, which represents a more sophisticated model than simply describing them as anti-inflammatory agents [4,14,30].

A further implication of this framework is that immune calibration may be understood not as passive attenuation of inflammation, but as active resetting of immune thresholds toward controlled responsiveness and resolution competence [34,35]. This distinction is pharmacologically relevant because therapies that recalibrate inflammatory set-points may differ fundamentally from those that merely suppress immune activation, particularly in conditions where excessive inflammation and immune paralysis can coexist [35,38,39]. Viewed in this light, postbiotics may have relevance not only as modulators of cytokine magnitude, but as regulators of immune tone, influencing balance, activation thresholds, and resolution programming in ways compatible with disease tolerance [34,35]. Such a perspective strengthens the argument that some postbiotic effects may be interpreted less as conventional anti-inflammatory activity and more as host-directed control of immunometabolic resilience [38,39]. Because inflammatory threshold setting is closely coupled to cellular redox state, this concept also reinforces the view that immune calibration and redox resilience may be mechanistically co-regulated rather than biologically separate processes [20,38,40].

Among the most rapidly expanding and genuinely high-interest areas in this field are microbial extracellular vesicles (EVs), precisely because they extend the discussion beyond metabolite biology into structured interkingdom signaling [41,42]. Their growing prominence is not incidental. EVs are attracting attention because they may function as biologically organized delivery systems carrying lipids, proteins, nucleic acids, and signaling cargo capable of influencing host pathways in ways free metabolites may not. That introduces a distinctly pharmacological dimension, since these vesicles can be interpreted as naturally evolved nano-scale carriers with potential relevance to target engagement and signal specificity [41,42,43]. Their importance to the present framework lies in emerging links to mitochondrial signaling, immunometabolic rewiring, and redox-sensitive host responses, all of which make them unusually compatible with the MIR-axis concept [44,45,46]. This leads to a provocative but increasingly plausible interpretation: if soluble metabolites represent one level of microbial signaling, extracellular vesicles may represent an emerging higher-order layer of microbial signaling architecture, raising the possibility that some postbiotic-associated extracellular vesicles may function as structured carriers of host-directed resilience signals, although these mechanisms remain incompletely defined [24,47]. However, much of this field remains mechanistically early, and translational relevance should presently be interpreted as hypothesis-generating rather than established.

## 3. Endothelium as an Underrecognized Pharmacologic Target

One of the more underdeveloped but potentially important ideas in the postbiotic field is that the endothelium may represent an underrecognized pharmacologic target rather than a downstream bystander of inflammation [48,49,50]. This distinction matters because endothelial dysfunction is increasingly recognized not simply as a consequence of critical illness, but as an active driver of permeability, microvascular dysregulation, thromboinflammation, and ultimately organ injury. In that context, the relevance of microbiome-derived metabolites extends beyond mucosal immunity or systemic inflammation alone. Emerging evidence suggests that metabolites such as short-chain fatty acids and indole derivatives may influence endothelial homeostasis through mechanisms linked to barrier integrity, oxidative signaling, and inflammatory tone [49,50,51,52]. This raises an important interpretive shift: if dysbiosis contributes to the loss of protective vascular signaling, then part of the therapeutic rationale for postbiotics may lie in restoring endothelial resilience, not merely modulating immune responses [48,51,53].

**Figure 2:**
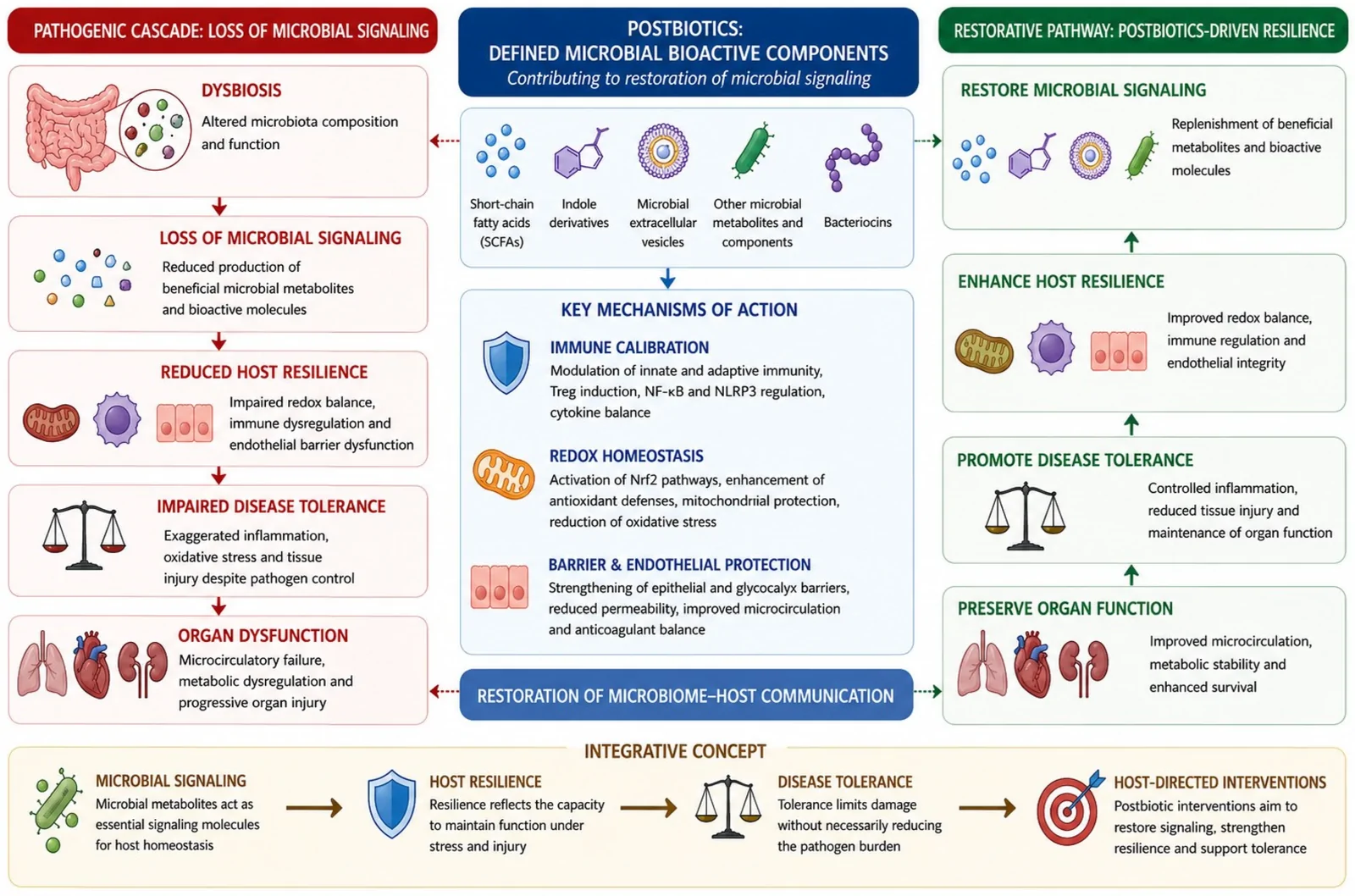
Conceptual framework illustrating how defined bioactive components contained in postbiotic preparations may contribute to restoration of microbial signaling, enhancement of host resilience, promotion of disease tolerance, and preservation of organ function through interconnected immune, redox, and endothelial pathways. The figure represents a hypothesis-generating conceptual model intended to illustrate potential host-directed intervention strategies rather than established therapeutic mechanisms. Solid arrows indicate the principal progression within each conceptual pathway, whereas dashed arrows represent conceptual interactions linking the pathogenic cascade, postbiotic-derived mechanisms, and restorative host responses. The lower panel summarizes the proposed integrative sequence linking microbial signaling, host resilience, disease tolerance, and host-directed interventions.

A particularly compelling layer of this concept centers on the endothelial glycocalyx, increasingly viewed as a vulnerable but biologically strategic interface where inflammation, coagulation, and redox injury converge. Glycocalyx disruption has been linked to capillary leak, leukocyte adhesion, platelet activation, and microthrombotic propagation, all central features of severe infection and organ dysfunction [54,55,56]. What makes this especially relevant for a postbiotic framework is the possibility that microbial signaling may influence glycocalyx preservation indirectly through redox buffering and inflammatory calibration. Short-chain fatty acids, particularly butyrate, are increasingly of interest in this regard, not only because of anti-inflammatory effects, but because emerging evidence suggests potential links to endothelial barrier support, oxidative stress modulation, and pathways plausibly relevant to glycocalyx stability, including Nrf2-associated cytoprotective signaling (Figure 2) [31,48,51,52,57]. While this area remains mechanistically incomplete, the implication is provocative: some postbiotics may contribute to vascular resilience at a level upstream of overt endothelial injury [48].

This is where biomarkers such as soluble thrombomodulin (sTM), von Willebrand factor (vWF), and D-dimer acquire more than prognostic significance; they may be interpreted as mechanistic readouts of endothelial injury and thromboinflammatory stress, and potentially as candidate pharmacodynamic markers for endothelial-targeted postbiotic interventions [58,59,60]. That distinction is important, because it reframes these biomarkers not solely as reflections of severity, but as tools that may help define endotypes in which endothelial-supportive postbiotic strategies are biologically rational (Table 3). Such an approach fits naturally with precision therapeutics, where biomarker-informed stratification increasingly shapes translational relevance.

This endothelial perspective also creates a conceptual bridge between microbiome biology and organ dysfunction that many reviews leave underdeveloped. Rather than viewing thrombosis, oxidative injury, and endothelial activation as parallel complications, they may be interpreted as interconnected manifestations of impaired host resilience [48,50,51,53]. In this framework, microbial metabolites, glycocalyx integrity, coagulation signaling, and organ function form a continuum rather than separate domains. That continuity is precisely what makes the endothelial axis a potentially powerful extension of the MIR concept, because it situates redox biology, immune calibration, and vascular protection within a shared systems-level model (Figure 2) [54,55,56,58,59,60]. Such a systems view also aligns with emerging network pharmacology concepts, in which therapeutic effects may arise from coordinated modulation of interconnected pathways rather than through single-target intervention [65,66].

Seen through this lens, postbiotics may be relevant not simply because they influence inflammation, but because they may help interrupt progression from endothelial stress to thromboinflammatory amplification and organ failure [54,55,56,57,58,59,60,65,66]. Although this hypothesis remains to be rigorously validated, it offers a pharmacologically testable framework linking restoration of microbial signaling to endothelial protection and organ resilience. Precisely because this framework bridges microbiome biology, vascular pathology, and host-directed therapeutics, it may represent one of the more translationally fertile directions in next-generation pharmabiotic research.

## 4. Precision Postbiotics in Critical Illness: A Translational Framework

**Figure 3:**
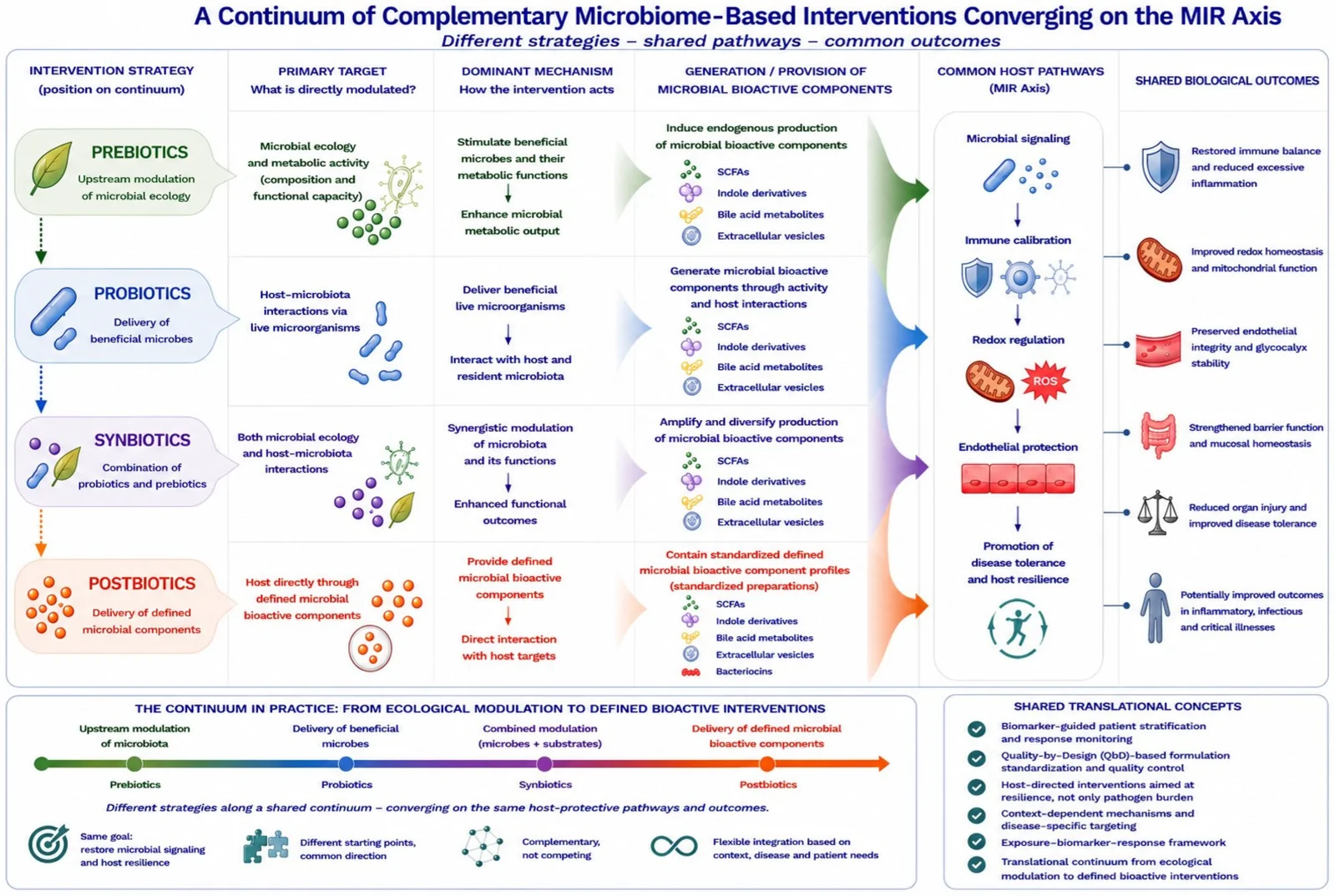
A continuum of complementary microbiome-based interventions converging **on the MIR Axis.** The figure illustrates a conceptual continuum of complementary microbiome-based intervention strategies, highlighting how prebiotics, probiotics, synbiotics, and postbiotics differ in their primary targets and mechanisms of action while converging on shared host-protective pathways. Although these interventions influence microbial ecology and host physiology through distinct upstream mechanisms, they ultimately promote overlapping biological processes—including microbial signaling, immune calibration, redox regulation, endothelial protection, and disease tolerance—represented by the MIR axis. Importantly, the microbial bioactive components shown (e.g., short-chain fatty acids, indole derivatives, secondary bile acid metabolites, extracellular vesicles, and bacteriocins) are presented as representative examples of molecules that may be generated through microbial activity or incorporated into defined postbiotic preparations, rather than as postbiotic classes themselves. The figure is intended as a hypothesis-generating conceptual model illustrating complementary, context-dependent translational strategies rather than hierarchical relationships among intervention classes. Vertical arrows indicate progression along the microbiome-based intervention continuum, colored arrows represent the dominant mechanistic flow toward the shared MIR axis pathways, and horizontal arrows illustrate convergence toward common biological outcomes.

Critical illness may represent one of the most compelling settings in which the therapeutic logic of postbiotics can be tested, precisely because dysregulated inflammation, oxidative injury, endothelial dysfunction, and loss of host resilience converge in a manner unlikely to be addressed by single-pathway interventions [50,51,53,54,58,59,60]. In this context, the relevance of postbiotics may lie less in conventional microbiome correction and more in their potential as metabolite-guided host-directed therapeutic strategies [65,66]. This reflects a broader paradigm shift from microbiome modulation toward microbial signaling therapeutics, where the objective is not primarily to alter community structure, but to restore functional pathways relevant to disease adaptation. The implication is important: in critical illness, where pathogenesis often reflects network-level failure rather than isolated molecular defects, multi-target signaling interventions may hold particular pharmacological interest (Figure 3) [11,12,13,14,15].

This perspective also helps explain why postbiotics may, in some contexts, offer advantages over live biotics. Reproducibility, formulation stability, and reduced safety concerns are often cited practical benefits, but their deeper importance is translational: they may allow microbial-derived interventions to be approached with a degree of standardization and mechanistic tractability closer to pharmacologic agents than ecological therapeutics [10,11,12,13,23,24,26]. That distinction becomes especially relevant in critically ill populations, where unpredictability of colonization, altered gut ecology, and safety concerns may limit the precision of live microbial approaches. Framed this way, the question is not whether postbiotics are universally superior to probiotics, but whether they may be better suited to settings where controllability and host-targeted signaling are therapeutically decisive [66,67].

Within the MIR framework, precision should not be viewed solely as a property of patient selection, but also as a property of postbiotic design. If microbial bioactive components constitute the functional drivers of metabolite signaling, immune calibration, and redox–endothelial resilience, then the biological activity of a postbiotic preparation is expected to depend on the qualitative and quantitative composition of these retained bioactive components. Consequently, precision postbiotics may originate not only from biomarker-guided therapeutic application but also from rational manufacturing strategies designed to reproducibly enrich specific microbial bioactive profiles [10,11,13,26].

From this perspective, microbial strain selection, fermentation substrate, cultivation conditions, growth phase, inactivation procedures, downstream processing, and formulation strategies become integral determinants of the final biological composition of postbiotic preparations. Rather than representing purely manufacturing variables, these processes collectively shape the repertoire of retained metabolites, extracellular vesicles, bacteriocins, and other microbial bioactive components that ultimately engage host pathways represented within the MIR axis [10,11,23,26].

This concept closely aligns with the principles of Quality by Design (QbD), in which predefined critical manufacturing parameters are systematically controlled to achieve reproducible product quality and biological performance [68,69,70,71]. Applied to postbiotics, such an approach could enable the rational enrichment of selected microbial bioactive components while preserving the preparation within the ISAPP consensus definition of a postbiotic, thereby linking manufacturing precision with mechanistic consistency and subsequent biomarker-guided patient stratification [10,11,26,68,69,70,71]. Viewed in this way, precision postbiotics represent the convergence of three complementary dimensions: precision manufacturing, precision biomarker characterization, and precision patient selection [10,11,24,26,65,66,67,68,69,70,71].

Building upon this manufacturing framework, a particularly provocative extension is the precision enrichment hypothesis, namely that postbiotic responsiveness may be biologically enriched within specific endotypes rather than uniformly distributed across patients [72,73]. This possibility matters because oxidative stress, endothelial injury, and inflammatory dysregulation are heterogeneous even within sepsis or severe viral illness [58,59,60]. It is therefore plausible that patients characterized by high oxidative burden, thromboinflammatory signatures, or barrier dysfunction may represent subgroups in which redox-active or endothelial-supportive postbiotics are mechanistically more relevant. Such a model aligns naturally with biomarker-guided precision medicine and shifts the field from generalized microbiome supplementation toward predictive enrichment strategies (Table 4) [72,73].

At the same time, the translational promise of this concept depends on unresolved questions being addressed explicitly rather than minimized. Dose definition, formulation-dependent bioactivity, biomarker-guided patient selection, and uncertainty regarding causal mechanisms remain central challenges [10,24,65,66,67,74]. Their importance is not a weakness of the field but part of what makes the pharmacological development of postbiotics credible rather than speculative. Indeed, it may be precisely the integration of mechanistic plausibility with these unresolved translational questions that makes postbiotics relevant to pharmacological research at this stage.

Taken together, postbiotics may be viewed not merely as successors to probiotics, but as promising candidates for microbiome-informed therapeutic interventions based on defined microbial bioactive components. Framed through the MIR axis, they offer a potential translational roadmap linking the restoration of microbial signaling to precision interventions in inflammatory and critical illness [24,66,72,74].

## 5. Current Challenges, Limitations and Future Perspectives

Despite growing enthusiasm, several limitations continue to constrain the translational development of postbiotics. Among the most important is the need for greater harmonization of terminology across scientific, regulatory, and commercial contexts. Although the ISAPP consensus statement has substantially advanced the field by providing a standardized definition of postbiotics, ongoing scientific and regulatory discussions remain regarding the scope of this definition, the classification of postbiotic preparations, and the distinction between postbiotic preparations and individual microbial bioactive components. Further harmonization of scientific terminology and regulatory frameworks will be essential to improve study comparability, product standardization, clinical translation, and regulatory approval pathways [10,11,24,26]. Ultimately, harmonized terminology and standardized product characterization represent fundamental prerequisites for reproducible manufacturing, meaningful biomarker interpretation, and successful clinical translation of postbiotic interventions.

Additional uncertainty surrounds formulation characteristics and exposure–response relationships, since pharmacologically meaningful dose ranges, bioactive component profiles, and delivery-dependent effects remain incompletely defined [23,24,39,43]. Addressing these challenges will require standardized manufacturing strategies guided by Quality by Design (QbD) principles, coupled with robust product characterization and biomarker-informed evaluation, to ensure reproducible postbiotic preparations suitable for translational development within a pharmabiotic or drug-like framework [68,69,70,71].

A second limitation concerns context dependence and potential unintended effects. Microbial-derived signals often exhibit pleiotropic and condition-sensitive behavior, raising the possibility that beneficial effects may vary across disease stage, host metabolic status, or inflammatory endotype. This is especially relevant for redox-active signaling, where hormetic principles suggest that adaptive versus adverse effects may depend on dose and biological context. Moreover, although postbiotics are often perceived as intrinsically safe, potential risks of off-target immunometabolic effects, maladaptive immune dampening, or unpredictable extracellular vesicle signaling remain insufficiently characterized and warrant caution [29,30,43,50,66].

An additional caution relates to safety liabilities that may accompany pharmacologic development, particularly for complex preparations such as extracellular vesicle-derived postbiotics. Potential concerns include immunogenic cargo effects, unintended perturbation of redox-sensitive signaling networks, or formulation-dependent toxicities that may not be apparent from current mechanistic studies [38,40,43,50,75]. Addressing these liabilities prospectively may be essential if postbiotics are to advance as credible host-directed therapeutics.

Another important limitation is causal uncertainty. Much of the evidence linking postbiotics to resilience, endothelial protection, or disease tolerance remains associative or mechanistically incomplete, and whether these mediators directly drive protection or instead reflect broader host–microbiome health often remains unresolved. This is particularly relevant to the MIR-axis and endothelial frameworks proposed here, which should be regarded as hypothesis-generating rather than clinically validated therapeutic models [48,53,54,55,56,57,58,59,60,65]. Recognition of these limitations does not weaken the field; rather, it defines the level of rigor needed for translation.

An additional translational challenge concerns regulatory classification and development pathways. Because postbiotics comprise heterogeneous preparations ranging from defined postbiotic preparations enriched in microbial bioactive components to complex extracellular vesicle-containing preparations and inactivated microbial preparations, their positioning within existing regulatory frameworks remains unsettled [11,24,26]. Depending on composition and intended use, some candidates may be viewed through the lens of functional biotics, whereas others may move closer to biologic or drug-like development paradigms. This ambiguity has implications for standardization, manufacturing quality control, pharmacodynamic characterization, and eventual clinical translation [11,24,26,68,69,70,71]. Importantly, this perspective should not be interpreted as implying that all postbiotic preparations should be regulated as pharmaceuticals. Rather, the appropriate regulatory pathway should remain proportionate to the composition, intended use, level of characterization, and supporting evidence for each specific preparation [11,24,26]. Clarifying these pathways while preserving appropriate regulatory flexibility may be essential if postbiotics are to realize their full potential across healthcare, nutrition, and other evidence-based applications [11,26].

These challenges also point toward future priorities. Progress will likely depend on greater compositional standardization, biomarker-guided patient stratification, and endotype-enriched testing to determine whether subsets characterized by oxidative stress burden or endothelial injury may be preferentially responsive to postbiotic interventions. In that sense, the central future implication is not whether postbiotics can replace existing biotics, but whether they can evolve into precision host-directed therapeutic strategies grounded in mechanistic pharmacology [65,66,72,73,74].

Future clinical studies should evaluate not only clinical outcomes, but also the quantitative relationship between defined postbiotic bioactive components and host pharmacodynamic biomarker responses, thereby establishing exposure–biomarker–response relationships analogous to those used in translational pharmacology [68,69,70,71,72]. Such an approach may facilitate biomarker-guided patient stratification, improve mechanistic understanding of treatment response, and support the rational development of standardized postbiotic interventions [11,24,68,69,70,71,72].

Collectively, these limitations define the key scientific and translational priorities that must be addressed before restoration of microbial signaling can evolve from a compelling biological concept into a reproducible, biomarker-guided clinical intervention (Table 5.).

## 6. Conclusions

Postbiotics are increasingly being viewed not simply as downstream products of microbial activity, but as candidates for standardized therapeutic interventions based on defined microbial bioactive components [10,11,24]. Framed through the MIR axis, their relevance may lie less in generalized microbiome modulation than in the possibility of restoring redox, immune, and vascular signaling pathways relevant to host resilience. This review advances the hypothesis that such effects may be particularly relevant in critical illness, where network-level dysfunction may favor multi-target, host-directed interventions over single-pathway approaches. Although major uncertainties remain regarding causality, standardization, dose definition, and biomarker-guided enrichment, these challenges are not simply obstacles to the concept, but part of its pharmacological maturation [11,24,68,69,70,71]. Viewed in this light, the future significance of postbiotics may depend less on replacing existing biotics than on whether they can evolve into credible precision therapeutics grounded in mechanistic pharmacology. Future validation of the MIR framework will require integration of mechanistic experimentation, biomarker-guided clinical studies, and multi-omics approaches capable of distinguishing causation from association.

## Figures and Tables

**Table 1:** Positioning of the proposed Metabolite–Immune–Redox (MIR) framework within existing conceptual models of microbiome–host interactions.

Conceptual Framework	Primary Focus	Major Contribution	Key Limitation	Additional Perspective Provided by the MIR Axis
Gut–Brain Axis	Bidirectional communication between the gut and central nervous system	Established the role of microbiota in neurological function and behavior	Primarily focused on neural and neuroendocrine signaling pathways	Expands the framework to include immune calibration, redox regulation, endothelial resilience, and biomarker-guided interventions
Immunometabolism	Interactions between metabolic pathways and immune function	Demonstrated how metabolism shapes immune responses	Limited emphasis on microbiome-derived signaling and vascular biology	Integrates microbial metabolites as upstream regulators of immunometabolic adaptation
Redox Biology	Oxidative stress and cellular redox homeostasis	Identified redox imbalance as a driver of inflammation and tissue injury	Usually investigated independently of microbiome signaling	Positions microbial-derived signals as modulators of systemic redox resilience
Host-Directed Therapy	Enhancement of host responses rather than direct pathogen targeting	Shifted therapeutic focus from pathogens to host resilience mechanisms	Does not specifically address microbial signaling pathways	Highlights the potential of postbiotics as microbiome-derived host-directed interventions acting through restoration of microbial signaling
Endothelial/Glycocalyx Models	Vascular integrity and microcirculatory homeostasis	Established endothelial dysfunction as a determinant of critical illness outcomes	Often disconnected from microbiome-centered mechanisms	Connects microbial signaling disruption with endothelial and glycocalyx injury
Precision Medicine	Individualized treatment based on biological heterogeneity	Promoted biomarker-guided patient stratification	Limited incorporation of microbiome-derived functional signals	Proposes biomarker-guided postbiotic interventions based on microbial signaling endotypes
Proposed MIR Axis	Integration of metabolite signaling, immune calibration, redox biology, endothelial resilience, and host-directed interventions	Provides a unified mechanistic and translational framework linking microbial signaling to host resilience	Requires further mechanistic validation and clinical testing	Integrates microbial metabolites, extracellular vesicles, immune responses, endothelial biology, redox adaptation, and biomarker-guided therapeutic strategies into a single framework

Footnote: Data synthesized and conceptually integrated from current literature on microbiome science, postbiotics, microbial metabolites, immunometabolism, redox biology, endothelial dysfunction, host-directed interventions, and disease tolerance [1,2,3,4,5,6,8,12,13,15,16,17,18,19,20]. The MIR framework is proposed by the authors as an hypothesis-generating integrative conceptual model and does not represent an established consensus classification.

**Table 2:** Comparative and context-dependent translational characteristics of prebiotics, probiotics, synbiotics, and postbiotics.

Feature	Prebiotics	Probiotics	Synbiotics	Postbiotics
Definition	Selective microbial substrates utilized by beneficial microbes	Live microorganisms conferring health benefit	Combination of probiotics and prebiotics	Non-viable microorganisms and/or their bioactive products
Predominant mechanisms of action	Selective modulation of microbiota composition and function	Live microbial modulation of host and microbiota interactions	Synergistic modulation of microbiota composition and host responses	Functional modulation through defined microbial bioactive components
Mechanistic specificity	Variable (substrate-dependent)	Variable (strain-dependent)	Variable (formulation-dependent)	Variable (potentially high for defined bioactive preparations)
Predominant therapeutic effect	Predominantly indirect	Direct and indirect	Integrated direct and indirect effects	Predominantly direct, with indirect effects depending on formulation
Dependence on host microbiome	High	Moderate–high	High	Generally lower
Standardization potential	Generally high	Variable	Variable	Potentially high
Manufacturing standardization potential	High	Variable	Variable	Potentially high
Dose precision	Formulation-dependent	Formulation-dependent	Formulation-dependent	Potentially high for standardized preparations
PK/PD tractability	Limited	Limited	Limited	Emerging (potentially high)
Target engagement definability	Variable (substrate-dependent)	Variable (strain-dependent)	Variable (formulation-dependent)	Potentially higher for defined bioactive preparations
Safety in critical illness	Favorable	Potential concerns	Potential concerns	Potentially favorable
Redox-modulating potential	Low–moderate	Moderate	Moderate–high	High
Precision medicine potential	Variable (substrate- and biomarker-dependent)	Variable (strain- and biomarker-dependent)	Variable (formulation- and biomarker-dependent)	Potentially high when supported by standardized preparations and appropriate biomarkers
Potential for biomarker-guided use	Variable (substrate-dependent)	Variable (strain-dependent)	Variable (formulation-dependent)	Potentially high for standardized bioactive preparations
Compatibility with host-directed therapy	Limited	Moderate	Moderate	High
Drug-like (“pharmabiotic”) properties	Minimal	Partial	Partial	Highest theoretical potential among current microbiome-based interventions *
Regulatory maturity	Moderate	Moderate	Limited	Emerging

Note: The characteristics summarized in this table are context-dependent and should not be interpreted as fixed or universal properties of each intervention class. They are intended to provide a comparative translational perspective and may vary according to formulation, purity, microbial strain or substrate, manufacturing quality, dose, biological target, host environment, regulatory oversight, quality-control procedures, and intended clinical application. Footnote: PK/PD, pharmacokinetics/pharmacodynamics. * pending mechanistic and clinical validation. Data synthesized from current consensus definitions and recent translational reviews [2,12,13,23,24,26,28,29,30].

**Table 3:** Candidate bioactive components of postbiotic preparations as precision redox interventions: mechanisms, translational relevance, and development challenges.

Bioactive Component of Postbiotic Preparations	Primary Molecular Targets	Redox Effects	Immune Calibration Effects	Endothelial Relevance	Candidate Pharmacodynamic Markers	Translational Caveats	Development Challenges
Short-chain fatty acids (e.g., butyrate)	GPCRs, HDAC inhibition, Nrf2-associated pathways	Oxidative stress buffering	Treg induction; inflammasome restraint	Barrier support; possible glycocalyx protection	IL-6, CRP, oxidative stress markers (e.g., MDA), syndecan-1 *	Dose/context dependence	Exposure-response uncertainty; formulation stability
Indole derivatives	AhR signaling; epithelial and immune pathways	Barrier-redox stabilization	Cytokine balancing; mucosal tolerance	Indirect vascular protection	AhR-responsive signatures; inflammatory cytokine profiles; barrier injury markers	Disease-stage dependence	Defined preparations needed
Secondary bile acid metabolites	FXR, TGR5 signaling	Metabolic redox regulation	Macrophage/cytokine modulation	Vascular-inflammatory and metabolic-endothelial effects	Metabolic-inflammatory signatures; exploratory endothelial biomarkers	Signaling pleiotropy	Narrow therapeutic window
Microbial extracellular vesicles (EVs)	Vesicle-associated proteins, lipids, nucleic acids	Mitochondrial/redox signaling	Immunometabolic rewiring	Emerging endothelial relevance	Cell-free DNA, syndecan-1 *, inflammatory EV cargo profiles **	Cargo heterogeneity	Purification, biodistribution, safety
Bacteriocins	Antimicrobial pathways; emerging host-signaling effects	Potentially indirect	Emerging anti-inflammatory effects	Mostly indirect	Not established	Limited translational evidence	Mechanistic validation needed

Note: In accordance with the ISAPP consensus definition, the entities listed in the first column represent candidate bioactive components that may be present within postbiotic preparations and should not be interpreted as postbiotic classes or as postbiotics themselves. Footnote: GPCR, G protein-coupled receptor; HDAC, histone deacetylase; Nrf2, nuclear factor erythroid 2-related factor 2; AhR, aryl hydrocarbon receptor; FXR, farnesoid X receptor; TGR5, G protein-coupled bile acid receptor 1; EVs, extracellular vesicles. Candidate PD markers are hypothesis-generating and not validated surrogate endpoints for postbiotic efficacy. * syndecan-1 as glycocalyx injury marker. ** EV cargo profiles = miRNA/protein signatures. Data synthesized from mechanistic and translational studies [6,20,21,31,32,33,36,37,41,42,43,44,45,46,47,48,52,53,54,55,56,57,58,59,60,61,62,63,64].

**Table 4:** Proposed biomarker-defined endotypes potentially responsive to postbiotic interventions.

Endotype	Biomarker Profile	Proposed Postbiotic Formulation	Mechanistic Rationale	Potential Pharmacodynamic Response Readouts
Hyperinflammatory phenotype	IL-6, CRP ↑	SCFA-enriched postbiotic formulations	Inflammasome restraint; immune calibration	IL-6/CRP reduction; attenuation of inflammatory signaling
Oxidative stress phenotype	MDA, F2-isoprostanes ↑	Redox-oriented postbiotic formulations	Nrf2-linked resilience support	Reduction in oxidative stress biomarkers; redox biomarker shift
Endothelial injury phenotype	vWF, sTM, D-dimer ↑	Barrier-supportive postbiotic formulations	Glycocalyx and vascular protection	Modulation of endothelial injury markers; exploratory syndecan-1 signal
Gut barrier dysfunction phenotype	Zonulin ^†^, LPS ↑	Indole-enriched postbiotic formulations	AhR-mediated barrier support	Reduction in permeability-associated markers; barrier function normalization

Footnote: Symbols: ↑ denotes an increase in the indicated parameter or risk. Abbreviations: CRP, C-reactive protein; MDA, malondialdehyde; sTM, soluble thrombomodulin; vWF, von Willebrand factor; LPS, lipopolysaccharide; AhR, aryl hydrocarbon receptor; SCFA, short-chain fatty acid. SCFA-enriched postbiotics refer to postbiotic preparations whose endogenous SCFA content has been enhanced through controlled strain selection, fermentation conditions, and manufacturing processes, rather than by addition of purified SCFAs. Similarly, indole-enriched postbiotic formulations refer to postbiotic preparations whose endogenous indole-derived bioactive component profile has been optimized through controlled microbial strain selection, fermentation, inactivation, and formulation processes, rather than by supplementation with purified indole compounds. ^†^ Zonulin should be interpreted cautiously as an exploratory surrogate of barrier dysfunction rather than a definitive permeability biomarker. Data synthesized from mechanistic and biomarker-enrichment literature [31,32,36,37,48,52,53,54,55,56,57,58,59,60,62,63,64,65,66,67,68,69,70,71,72,73,74].

**Table 5:** Outstanding research priorities for translating microbial signaling restoration into biomarker-guided clinical practice.

Research Area	Current Knowledge Gap	Translational Importance
SCFA Signaling	Which microbial bioactive components are causally responsible for protective immune and endothelial effects?	Identification of therapeutic targets and exposure–response relationships
Extracellular Vesicles	Which vesicle cargo molecules mediate biological activity and tissue-specific signaling?	Development of next-generation postbiotic therapeutics
Endothelial and Glycocalyx Biology	Can microbial signaling restoration preserve endothelial function and glycocalyx integrity in humans?	Translation into critical care and inflammatory disease interventions
Biomarker-Guided Therapy	Which biomarkers best identify individuals most likely to respond to postbiotic interventions?	Precision medicine and patient stratification
Exposure–Response Relationships	What concentrations and durations of postbiotic exposure are required for clinically meaningful effects?	Rational dosing and therapeutic optimization
MIR Framework Validation	Can restoration of microbial signaling be quantitatively measured and linked to clinical outcomes?	Validation of the MIR axis as a translational framework
Multi-Omics Integration	Which microbial pathways demonstrate causal rather than associative relationships with disease outcomes?	Identification of actionable mechanisms and therapeutic targets
Quality by Design and Manufacturing	Which manufacturing parameters reproducibly determine the qualitative and quantitative composition of retained microbial bioactive components?	Standardized postbiotic manufacturing, reproducible product quality, and regulatory translation

Footnote: Data synthesized from recent reviews and translational studies addressing postbiotics, microbial bioactive components, extracellular vesicles, endothelial dysfunction, biomarker-guided precision medicine, host-directed interventions, Quality by Design, and microbiome-based interventions [2,3,4,5,11,12,15,38,43,45,50,62,63,64,65,66,67,68,69,70,71]. The listed priorities represent key unresolved questions identified across the current literature and integrate current concepts in postbiotic biology, Quality by Design, biomarker-guided precision medicine, and the MIR framework to guide future mechanistic and clinical research.

## Data Availability

No new data were created or analyzed in this study. Data sharing is not applicable to this article.

## Abbreviations

The following abbreviations are used in this manuscript:

AhR	Aryl Hydrocarbon Receptor
CRP	C-Reactive Protein
D-dimer	D-dimer
EVs	Extracellular Vesicles
FXR	Farnesoid X Receptor
GPCR	G Protein-Coupled Receptor
HDAC	Histone Deacetylase
IL-6	Interleukin-6
LPS	Lipopolysaccharide
MDA	Malondialdehyde
MIR	Metabolite–Immune–Redox
MISEV	Minimal Information for Studies of Extracellular Vesicles
NLRP3	NLR Family Pyrin Domain Containing 3
Nrf2	Nuclear Factor Erythroid 2–Related Factor 2
PD	Pharmacodynamic
PK/PD	Pharmacokinetics/Pharmacodynamics
ROS	Reactive Oxygen Species
SCFAs	Short-Chain Fatty Acids
sTM	Soluble Thrombomodulin
TGR5	Takeda G Protein-Coupled Receptor 5 (G Protein-Coupled Bile Acid Receptor 1)
Treg	Regulatory T Cell
vWF	von Willebrand Factor
